# DNA damage in oral mucosal epithelial cells cultured in complex and xenobiotic-free media: a comparison study

**DOI:** 10.1093/mutage/geaf008

**Published:** 2025-06-28

**Authors:** Joao Victor Cabral, Sona Vodenkova, Kristyna Tomasova, Ludmila Vodickova, Naouale El Yamani, Elise Rundén Pran, Maria Dusinska, Adam Safanda, Katerina Jirsova

**Affiliations:** Laboratory of Biology and Pathology of the Eye, Institute of Biology and Medical Genetics, 1st Faculty of Medicine and General Teaching Hospital, Charles University, Czech Republic; Department of Molecular Biology of Cancer, Institute of Experimental Medicine of the Czech Academy of Sciences, Prague, Czech Republic; Biomedical Centre, Faculty of Medicine in Pilsen, Charles University, Pilsen, Czech Republic; Department of Molecular Biology of Cancer, Institute of Experimental Medicine of the Czech Academy of Sciences, Prague, Czech Republic; Biomedical Centre, Faculty of Medicine in Pilsen, Charles University, Pilsen, Czech Republic; Biomedical Centre, Faculty of Medicine in Pilsen, Charles University, Pilsen, Czech Republic; Institute of Biology and Medical Genetics, 1st Faculty of Medicine, Charles University, Prague, Czech Republic; Health Effects Laboratory, Department of Environmental Chemistry and Health Effects, NILU- The Climate and Environmental Research Institute NILU, 2007 Kjeller, Norway; Health Effects Laboratory, Department of Environmental Chemistry and Health Effects, NILU- The Climate and Environmental Research Institute NILU, 2007 Kjeller, Norway; Health Effects Laboratory, Department of Environmental Chemistry and Health Effects, NILU- The Climate and Environmental Research Institute NILU, 2007 Kjeller, Norway; Department of Pathology, First Faculty of Medicine, Charles University and General University Hospital, Prague, Czech Republic; Laboratory of Biology and Pathology of the Eye, Institute of Biology and Medical Genetics, 1st Faculty of Medicine and General Teaching Hospital, Charles University, Czech Republic

**Keywords:** oral mucosal epithelial cells, limbal stem cell deficiency, genomic stability, micronucleus test, comet assay

## Abstract

In this study, we evaluated the genomic stability of oral mucosal epithelial cells (OMECs) cultured in complex media (COM) and xenobiotic-free media (XF) to assess their potential clinical application for limbal stem cell deficiency (LSCD) treatments. OMECs serve as a promising autologous cell source for bilateral LSCD treatment, offering an alternative to limbal epithelial cells (LECs). However, genomic integrity is crucial to ensure the long-term success of transplanted cells. We performed micronucleus (MNi) tests and comet assays to compare DNA damage in OMECs cultured in both media types. The results indicated no significant differences in cell morphology, viability, or size between the two conditions. The MNi frequency was similar, with 5.67 and 6.17 MNi per 1,000 cells in COM and XF conditions, respectively. Comet assay results showed low levels of strand breaks (SBs) and oxidized DNA lesions in both media, with XF showing a slightly lower, albeit statistically insignificant, percentage of tail DNA for net Fpg-sensitive sites. Our findings suggest that OMECs can be effectively cultivated in either COM or XF media without inducing significant DNA damage, supporting the potential use of XF media in clinical settings to reduce contamination risks. This study underscores the importance of genomic stability in cultured cells for ocular surface transplantation, contributing valuable insights into optimizing culture conditions for safer and more effective clinical applications.

## Introduction

Regenerative medicine using cultured epithelial cell grafts offers an attractive therapy for various epithelial disorders, such as unilateral or bilateral corneal limbal stem cell deficiency (LSCD). The disease is caused by the destruction or dysfunction of limbal epithelial stem cells (LESCs) located in the limbus, a tissue that surrounds the cornea. LESCs are essential for corneal epithelial regeneration. The corneal epithelial cells that are physiologically shed or die as a result of various pathologies are replaced by LESCs that differentiate and migrate centripetally to the corneal epithelium [1]. When LESCs are destroyed by injury, such as chemical or thermal burns, or are absent as a result of genetic disease, their replacement by stem cell-containing tissue or by stem cells is the only effective therapy [2].

Unilateral LSCD can be treated by direct transplantation of limbal tissue derived from a healthy eye or by a cell graft composed of cultured limbal epithelial cells (LECs) containing stem cells [3]. Besides LECs, there are also oral mucosal epithelial cells (OMECs), by far the most studied autologous cell source in treating the bilateral form of LSCD [4]. The main adverse effect of using OMECs is neovascularization of the cornea, which occurs gradually in most cases, usually during the first year after *ex vivo* cultivated OMECs transplantation [5,6]. The treatment of LSCD using autologous cell transplantation minimizes the risk of allograft rejection, and in the case of first graft failure, it can be repeated [7,8].

The cultivation of human (stem) cells for transplantation in the presence of growth-promoting xenobiotics of animal (fetal calf serum, animal-derived growth factors) or human origin represents a major safety concern because it can cause adverse reactions (risk of pathogen transmission, development of tumorigenic effect, immune reaction, or graft rejection) after clinical applications [9–11]. The increase in the number of cell transplants necessitates improvements in current processes and the development of new standards and protocols, particularly in terms of transplant safety. Thus, the worldwide trend to minimize adverse effects after cell transplantation leads to the cultivation of cells with the lowest possible amount or even absence of xenobiotic components [12,13]. This approach is also often used for the preparation of OMECs and LECs.

For both LECs and OMECs, two main types of media are generally used. The more efficient complex (COM) media contain, besides the main medium, additives like growth factors and supplements supporting cell attachment, growth, and proliferation capacity [4,14]. On the other hand, xenobiotic-free (XF) media, in which additives can be advantageously substituted by the serum of the patient for whom the cell graft is being prepared [5,15,16], are safer in terms of the occurrence of the patient’s adverse effects.

The composition of the media and other factors like culture duration, storage temperature, and the presence of cell substrates directly influence the genome stability, oxidative damage levels, and the effectiveness of antioxidant defense and repair mechanisms in cultured cells [17–19]. Maintaining integrity of the DNA is a prerequisite for the proper long-term functioning of the cell graft, cell replication, and viability in the generated tissue [20]. Therefore, the measurement of DNA damage, and particularly oxidative DNA damage (e.g. 8-oxoguanine), may provide information regarding the quality of a cell culture system.

The genomic instability of cultured proliferating cells can be analyzed by several approaches. The micronucleus (MN) test detects permanent genetic damage—clastogenicity and aneugenicity—serving as one of the key methods to detect genotoxicity or changes arising from aberrant proliferation [21,22]. The comet assay measures transient DNA lesions, such as strand breaks (SBs) or oxidative DNA lesions (by a modified version with the inclusion of DNA repair enzymes) [23]. Both tests are used for mapping nuclear DNA damage in individual cells, which is consistent with the need to detect potential DNA damage in a growing culture [24].

Both the MN test and comet assay have been applied to epithelial cells of various origins, including the nasal epithelium, conjunctival epithelium, or buccal mucosa [25–27], which are the cells of our interest. The oral mucosal epithelium has also been used for detecting not only SBs but also more specific DNA damage, such as lesions caused by DNA oxidation [23]. Detecting genotoxicity and assessing DNA damage in cultured cells is crucial to guarantee the safety and effectiveness of ocular surface transplantation. This is because the long-term regeneration of the post-transplant surgery epithelium greatly depends on the availability of a functional reservoir of stem cells [20].

We aimed to detect potential DNA damage in highly proliferating OMEC cultures in conditions that could be transferable to clinical practice. Two culture systems using either COM media or XF media were tested and compared for their safety in terms of genomic integrity.

## Material and methods

### The oral mucosal epithelial cell sampling

The retrieval of oral mucosal donor tissue adhered to the project guidelines titled “Experimental graft preparation for the treatment of limbal stem cell deficiency from oral mucosal cells” and local ethical approval (IS, 1041/19 S-IV). The study followed the principles outlined in the Helsinki Declaration. The collection of donor tissue complied with all legal requirements in the Czech Republic, including the condition that the donor will not be listed on the national register of individuals opposing the post-mortem removal of tissues and organs. Informed consent is not mandated for using donor tissue under Czech law (Law Act No. 372/2011 Coll.) if the data are anonymized before being entered into the record.

Seven explants were procured from the Department of Pathology, General Teaching Hospital, and the First Faculty of Medicine, Charles University, Prague, Czech Republic. The explants included two female donors (80 ± 5.6 years old, mean ± SD) and five male donors (70.2 ± 14 years old, mean ± SD), with an average post-mortem time of 27.9 ± 10.7 hours until tissue retrieval. Causes of death included cardiorespiratory failure (five cases), hepatic failure (one case), and brain hemorrhage (one case).

Oral mucosa tissue was sampled inside the mouth, 20 mm behind the angle of the mouth. Just before the sampling, the collection site was treated with 10% iodinated povidone (Betadine, EGIS, Pharmaceuticals, Budapest, Hungary) diluted in 0.9% NaCl (B. Braun, Melsungen, Germany) for 1 minute. A superficial rounded incision was made using a disposable and sterile 6 mm biopsy punch (Kai Medical, Seki City, Japan) and removed with a scalpel. Two samples were collected from the right and left buccal mucosal side and preserved in BASE 128 (Alchimia, srl., Ponte San Nicolò, Italy) at 4°C until further processing.

### Preparation of cell suspension

The donor’s oral mucosa samples were rinsed three times in BASE 128 (Alchimia) for 10 minutes each, followed by three washes in phosphate-buffered saline (PBS), with each wash lasting 3 minutes. Subsequently, each sample was halved and collectively immersed in 6 ml of dispase II solution (Gibco, New York, NY, USA) at a concentration of 1 U/ml and incubated at 37°C for 1 hour. After the enzymatic treatment, the samples were transferred to PBS, and the epithelium was separated from the submucosa by tweezers. The epithelial samples were then transferred in 1.5 ml of 0.05% trypsin- ethylenediaminetetraacetic acid (EDTA) (Gibco, New York, NY, USA) and incubated for 15 min. The epithelial cells were then harvested using a cell scraper (Corning, New York, NY, USA). The trypsin-EDTA was neutralized by diluting with 3 ml of Dulbecco’s Modified Eagle’s Medium (DMEM)-F12 media containing 20% of human serum (HS). The resulting cell suspension was filtered through a 70-μm cell strainer (PluriSelect, Leipzig, Germany) and centrifuged for 10 minutes at 250 × g in a Universal 32 R centrifuge (Hettich Zentrifugen, Tuttlingen, Germany). The supernatant was discarded, and the cell pellet was resuspended in 1 ml of DMEM-F12 media. Cell counting was performed by diluting the cells in a 1:1 ratio with Trypan Blue dye 0.4% (Bio-Rad, Hercules, CA, USA) using the TC20 Automated Cell Counter (Bio-Rad, Hercules, CA, USA).

### Cell seeding and culture

The fibrin sealant (Tisseel Lyo, Baxter, Zurich, Switzerland) was prepared following the manufacturer’s instructions, as described in detail [15]. Per each well of a 24-well plate (VWR, Radnor, PA, USA), 300 μl of fibrin sealant was used. Cells were seeded at a concentration of 4.5 × 10^4^ cells (in about 200 µl media) onto the fibrin gels, equating to approximately 2.36 × 10^4^ cells/cm^2^ (i.e. 45,000 cells/well).

The following media were used: COM medium, comprising Dulbecco’s Modified Eagle Medium/Nutrient Mixture DMEM/F12 (1:1) with GlutaMAX (Gibco, New York, NY, USA) 10% pooled HS, 1% antibiotic-antifungal solution (Gibco, New York, NY, USA), 10 ng/ml recombinant epidermal growth factor (EGF) (Gibco, New York, NY, USA), 5 µg/ml insulin-transferrin-selenium (Merck KGaA, Darmstadt, Germany), 0.4 µg/ml hydrocortisone (VUAB Pharma A.S., Roztoky, Czech Republic), 24 µg/ml adenine hydrochloride (Merck KGaA, Darmstadt, Germany), 1.4 ng/ml triiodothyronine (Merck KGaA, Darmstadt, Germany), and 8.4 ng/ml cholera toxin (Merck KGaA, Darmstadt, Germany); XF medium was supplemented with 10% HS and 1% antibiotic-antifungal solution. Both media were supplemented with 160 μl/ml tranexamic acid (Merck KGaA, Darmstadt, Germany) to prevent the digestion of the fibrin gel in the cell culture.

Human blood, approximately 40 ml, was drawn from the antebrachial vein of seven volunteers at the local transfusion department (General University Hospital in Prague, Prague, Czech Republic). All donors were tested negative for hepatitis B and C, syphilis, and HIV, with C-reactive protein levels below 20 mg/L. The HS was prepared as described before [28] without dilution, pooled from seven donors, and stored at −20°C until use.

The culture medium was replaced daily during the initial week and then every other day until reaching confluence. Throughout the culture period, the cells underwent continuous monitoring for proliferation, confluence, and morphological changes. This assessment was conducted using an inverted phase-contrast microscope Olympus CKX41 (Olympus, Tokyo, Japan) equipped with an EOS 250D camera (Olympus, Tokyo, Japan). Images were captured using QuickPhoto Camera software (Promicra, Prague, Czech Republic), see **Supplementary Material 1**.

### Harvesting cultured cells

Upon reaching 85 to 95% confluence, cells were harvested for the MN test and the comet assay. The harvesting procedure was carried out as follows: (i) The wells were washed twice with 500 μl of dispase II (1 U/ml). (ii) 1 ml of dispase II (1 U/ml) was added, and the fibrin gel was separated from the plastic plate using tweezers. The gel was then chopped into smaller pieces with surgical ophthalmic scissors and incubated for 30 minutes at 37°C. (iii) The solution was transferred to a 15-ml centrifuge tube and centrifuged for 10 minutes at 250 × g. (iv) The supernatant was removed, and the cell pellet was resuspended in 2 ml of TrypLE Express (Gibco, New York, NY, USA) and incubated for 25 minutes. (v) 4 ml of DMEM-F12 media was added to halt the enzymatic activity, followed by centrifugation for 10 minutes at 250 × g. (vi) The supernatant was removed, and the cell pellet was resuspended in 1 ml of DMEM-F12 media. (vii) The cell concentration was determined as described above.

### Micronucleus test

For the MN test, cytospin slides were prepared by spinning cells at 180 × g for 5 minutes, and the slides were left to air dry overnight. The next day, cells were fixed in 300 μl of 4% paraformaldehyde (Fluka Chemical, Buchs, Switzerland) for 15 minutes at room temperature. Afterward, the slides underwent three washes in PBS for 5 minutes each. Finally, the slides were stored in a Coplin jar with PBS at 4°C until needed.

DNA was visualized using fluorescent staining with 4,6-diamidino-2-phenylindole dihydrochloride (DAPI, Invitrogen, Rockford, USA) diluted in PBS 1:5000 [29,30]. Cells were mounted in Mowiol (Aldrich, Karlsruhe, Germany) to prevent the decrease of fluorescent signal.

Micronuclei (MNi) were evaluated according to the criteria for MNi determination [31,32] except that the cytokinesis block was not used. Only cells that were not clumped or overlapped with undamaged round intact cell nuclei were included in the MN analysis. Smooth round or oval nuclear fragments smaller than 1/3 of the main nucleus, that could be distinguished from artifacts, and visibly separated from the main nucleus were classified as MNi. MNi also exhibited the same intensity staining, texture, and refraction as the main nucleus. Cells undergoing visible degenerative processes, such as apoptosis (nuclear fragmentation), karyorhexis, and karyolysis, were not involved in MNi calculation, similarly to cells with doubtful MNi-like structures were not included in the assessment [31,32].

A minimum of 1,000 epithelial cells with intact nuclei and well-preserved cytoplasm were evaluated independently by two experienced observers (KJ and VV) using previously described criteria [32]. The final score was calculated as the average of their assessments. MNi were analyzed using a Nikon Eclipse Ni-U H600L microscope at × 400 and × 1000 magnification, and images were captured with a Nikon DS-Fi3 camera (both from Nikon Corporation, Tokyo, Japan). MNi frequency was expressed as the number of MNi per 1,000 epithelial cells (‰).

### Comet assay

For the comet assay, samples (as detailed in “Harvesting cultured cells”) underwent centrifugation once more for 5 minutes at 700 × g, at 4°C, and then they were washed in 1 ml of PBS (2–8°C).

After spinning the samples again and removing the supernatant, the pelleted cells were embedded in the appropriate volume of 0.5% low melting point agarose (LMP) dissolved in PBS (both Merck KGaA, Darmstadt, Germany) to achieve a final concentration of 2 × 10^5^ cells/ml. From each LMP-cell suspension mixture, two 70 μl drops were transferred to each microscope slide precoated with 1% normal melting point agarose in distilled water, resulting in an approximate final cell count of 14,000 cells per gel. For each sample, two parallel slides containing two gels were prepared, one for incubation with buffer B and the other for incubation with formamidopyrimidine-DNA glycosylase (Fpg, New England of Biolabs, MA, USA). Additionally, assay controls (light-treated and Ro19-8022 (Hoffman-La Roche Ltd., Basel, Switzerland) plus light-treated HCT-116 cells) were included in each experiment to verify the method’s performance. The gels containing cells were covered with 22 × 22 mm coverslips and left to sit for 5–10 minutes at 4°C. Following this, the coverslips were removed, and the slides were placed into a lysis solution in a Coplin jar. The cells were lysed in a solution consisting of 2.5 M NaCl, 0.1 M Na_2_EDTA, and 10.0 mM Trizma base with 1% Triton X-100 (pH 10) at 4°C for 2.5 hours, with the standard time applied consistently across all experiments. After finishing lysis, slides were washed three times with buffer B (40 mM HEPES, 0.5 mM Na_2_EDTA, 0.2 mg/ml BSA, 0.1 M KCl, pH 8, 4°C) for 5 minutes at 4°C using a Coplin jar. Subsequently, each sample was either incubated with buffer B or Fpg enzyme (8.000x diluted in buffer B) at 37°C for 30 minutes.

The microscopic slides were then transferred to the electrophoresis tank containing electrophoresis solution (0.3 M NaOH, 1 mM Na_2_EDTA, 4°C), incubated for 40 minutes at 4°C in the dark, and then electrophoresed at 1.15 V/cm for 30 minutes at 4°C in the dark. Following electrophoresis, slides were neutralized in PBS for 10 minutes at 4°C and then washed in distilled water for 10 minutes at 4°C. Slides were left to dry until the next day when they were stained with SYBR Gold (Thermo Fisher Scientific, Waltham, MA, USA), visualized with a fluorescence microscope Olympus BX63 (Olympus, Tokyo, Japan), and analyzed using semi-automated Lucia Comet Assay™ software (Laboratory Imaging, Prague, Czech Republic). One hundred comets were scored per gel (i.e. two hundred comets per slide). Median tail intensity (TI; % tail DNA) per gel and the mean TI of replicate gels were used as parameters to describe the comets. Fpg-sensitive sites were calculated as Net Fpg-sensitive sites using this formula: *Fpg-sensitive sites = TI*_*Fpg*_*—TI*_*Buffer*_. All the steps in the alkaline comet assay used in this study adhered to MIRCA recommendations [33] and followed the previously published protocols [23,34].

### Statistical analysis

As the sample size is small to assume a normal distribution, a nonparametric test, the Mann–Whitney *U* test, was employed. The data were analyzed using StatPlus:mac (AnalystSoft Inc. - statistical analysis program for macOS, version v8) and Microsoft Excel for Mac (v. 16.75). All *P*-values were two-tailed, with *P* < .05 considered statistically significant (significance levels were denoted as follows: *P < .05; **P < .01; ***P < .001; ****P < .0001).

## Results

**Figure 1.**
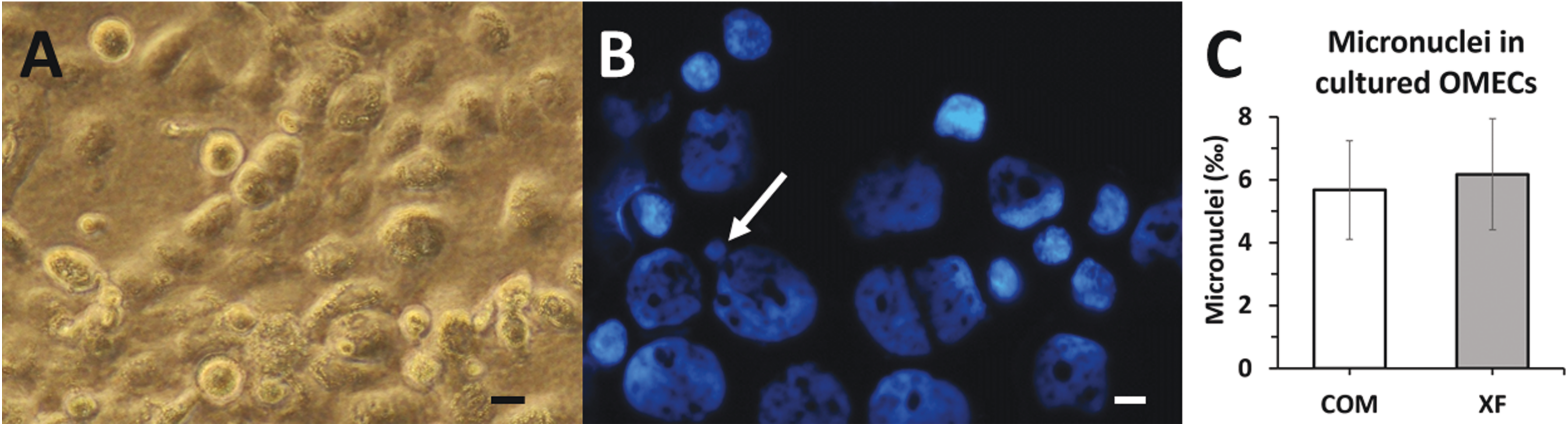
Oral mucosal epithelial cells (OMECs). (A) Inverted phase-contrast microscopy of OMECs cultured on fibrin in complex medium, showing the cell proliferation after 10 days of culture. (B) The micronucleus (arrow) in harvested and cytospined OMECs. Nuclei are counterstained with DAPI. Scale bars: 10 μm. (C) Quantification of the number of micronuclei in OMECs cultured in complex (COM) or xenobiotic-free (XF) media.

The genotoxicity assays were performed in seven culture pairs (COM and XF) within an average of 13.7 ± 1.0 (mean ± SD) cultured days. OMECs cultured in COM media (**Fig. 1A**) reached confluence on average about one day earlier. There were no differences between both conditions regarding cell morphology, viability, and size.

The number of MNi reached 5.67 ± 1.45 and 6.17 ± 1.64 per 1,000 cells in OMECs cultured in COM or XF conditions, respectively, with no statistically significant difference (*P* = .59). MNi were discernible from the main nucleus and were all smaller than 1/4 of the nucleus, see **Fig. 1B**.

**Figure 2.**
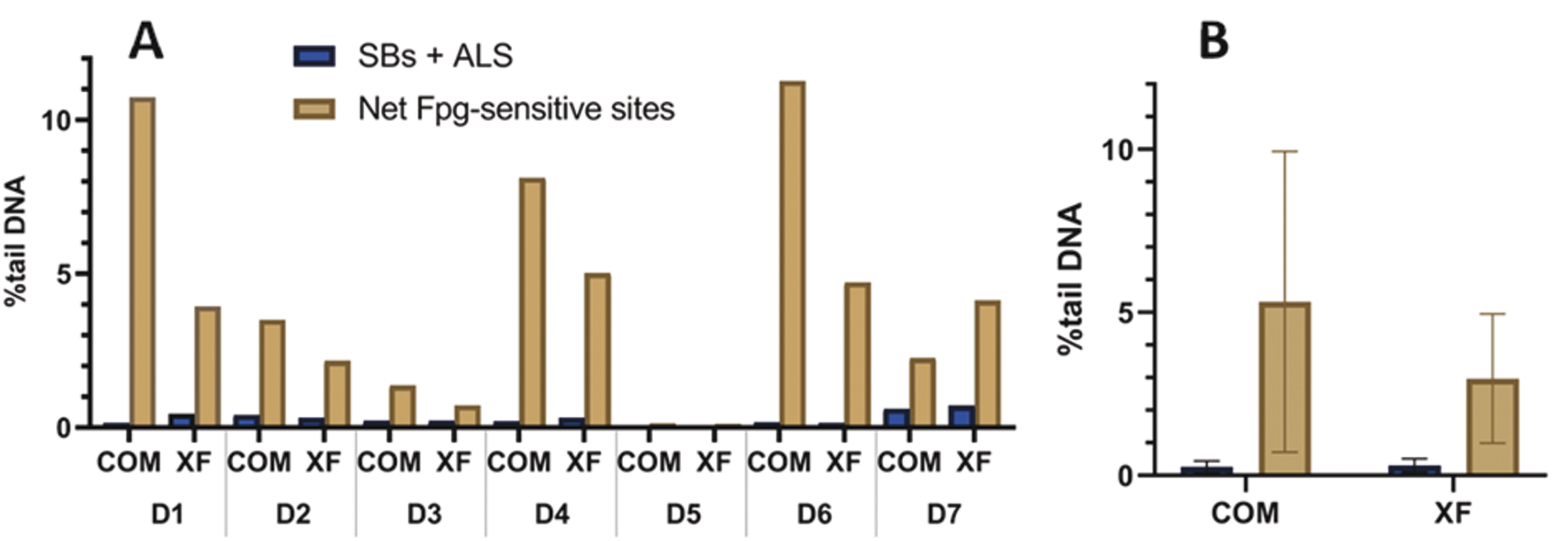
The mean (B) individual (A) values of DNA damage (SBs + ALS) and net Fpg-sensitive sites, expressed as % tail DNA, were detected in seven samples of oral mucosa cells from donors D1-7 expanded *ex vivo* in either COM or XF media. The net Fpg-sensitive sites were calculated from the sum of SBs + ALS and Fpg-sensitive sites minus the level of SBs + ALS. ALS, alkali-labile sites; COM, complex; D, donor; Fpg, formamidopyrimidine-DNA glycosylase; SBs, strand breaks; XF, xenobiotic-free.

**Figure 3.**
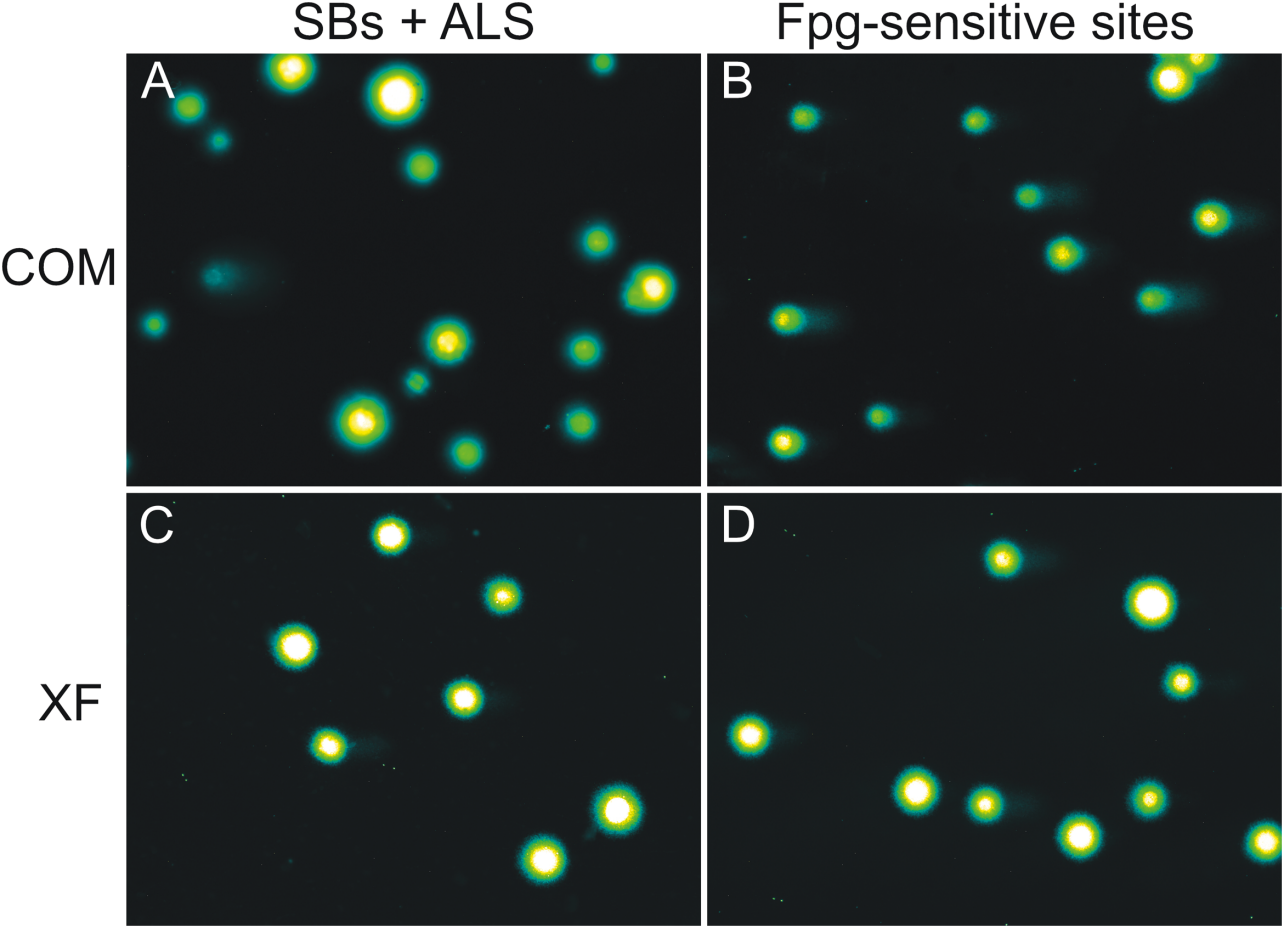
DNA damage in oral mucosal epithelial cells from donor D7 cultured in either COM or XF media. For SBs, including ALS, we present the results in parts A and C. To detect oxidized purines, we incorporated a digestion process using the bacterial DNA repair enzyme Fpg, with these results shown in parts B and D. ALS, alkali-labile sites; COM, complex; Fpg, formamidopyrimidine-DNA glycosylase; SBs, strand breaks; XF, xenobiotic-free.

OMECs cultivated in both COM and XF media showed very low levels of DNA SBs + ALS after 2 weeks of culture. Similarly, the levels of net Fpg-sensitive sites were low for both types of cell media, with a slight but not statistically significant decrease observed in the XF medium. In cells cultured in COM medium, the % tail DNA was 0.26 ± 0.18 (mean ± SD) for SBs + ALS and 5.33 ± 4.62 (mean ± SD) for net Fpg-sensitive sites. On the other hand, in cells cultured in XF medium, the % tail DNA was 0.31 ± 0.20 (mean ± SD) for SBs + ALS and 2.96 ± 1.98 (mean ± SD) for net Fpg-sensitive sites, see **Fig. 2B**. Individual sample data is shown in **Fig. 2A**. The representative images of the nucleoids from donor D7 produced by the comet assay and visualized by a fluorescent microscope are shown in **Fig. 3**. There was no statistically significant difference (Mann–Whitney *U* test) between SBs + ALS (*P* > .05) or net Fpg-sensitive sites (*P* > .05) in COM medium compared to XF medium.

## Discussion

In the context of ocular surface transplantation, analyzing genomic stability in cultured cells is of paramount importance for ensuring the safety and efficacy of the procedure. The sustained regeneration of the epithelium post-grafting heavily relies on a functional reservoir of stem cells [20,35].

For oral mucosal epithelium, cultured cells lose their natural niche and thus the long-term homeostasis. Additionally, when employing *ex vivo* systems that introduce a foreign microenvironment, cellular functionality can undergo temporary or permanent alterations due to oxidative reactions and other stressors. As a result of the loss of intercellular connections, aberrant proliferation may also occur. All these changes have the potential to compromise the integrity of cellular molecules, thus predisposing them to damage [19]. Consequently, our study aimed to assess genomic stability by investigating potential and transient DNA damage in cultured OMECs.

As a rapid screening analysis, the MN test without a cytokinesis blocker was chosen to assess chromosomal aberrations due to clastogenicity [36]. Our results did not indicate the significant differences in OMECs cultured in COM and XF conditions. The number of MNi reflects the high proliferation potential of cells in both culture media. The relatively higher levels of micronuclei (5–9 MNi/1000 cells) are likely due to the fast OMEC proliferation under cell cultivation. Similar values have already been observed in the culture of primary nasal epithelial cells [37] or epithelial cell lines [38].

Using the comet assay, no statistically significant difference was observed between the levels of SBs levels combined with ALS or net Fpg-sensitive sites in cells cultured in COM or XF media. Although the results were not statistically significant, net Fpg-sensitive sites consistently exhibited higher levels of % tail DNA compared with SBs + ALS in both COM and XF conditions. Fpg-sensitive sites were more frequently detected in COM conditions, though the difference was not statistically significant. The occurrence of the Fpg-sensitive sites in cells cultivated in COM media was especially noticeable in two subjects, where the % tail DNA exceeded 10%, but still remained within the borderline of normal background level distribution (12%) of DNA damage [39].

These discrepancies imply that COM and XF media may exert slightly different effects on DNA stability in OMECs, necessitating further exploration into the underlying mechanisms. Our findings align with previously reported data by Baricevic et al. [40], who demonstrated through an alkaline comet assay that buccal cells from control samples (subjects who had been without teeth for at least 5 years) exhibited a range of % tail DNA of 0.36 ± 1.19 (mean ± SD). In our study, cultured cells in COM medium exhibited % tail DNA of 0.26 ± 0.18 (mean ± SD) for SBs + ALS, while those in XF medium showed 0.31 ± 0.20 (mean ± SD). This indicates that our cultured cells do not harbor higher DNA damage compared to non-cultured cells. However, their study did not evaluate Fpg-sensitive site damages, which are indicative of oxidative DNA lesions.

Conversely, our findings for OMECs contrast with those of Lorenzo et al. [20], who compared LECs expanded in COM media and media containing only HS as an additive. This study revealed substantial SB levels but relatively low levels of net Fpg-sensitive sites in LECs cultivated in either medium. Nonetheless, in both—ours and Lorenzo´s studies, both types of damage remained relatively low. Despite the relative simplicity of XF, we did not observe any elevation of oxidized bases in DNA. Previous research suggests that cells cultured in a medium supplemented with HS may exhibit enhanced genome stability and the ability to maintain an unmethylated state compared to those cultured in media supplemented with fetal bovine serum [17,41]. It is noteworthy to emphasize that pooled HS was used in both our culture conditions.

Cells were cultured on fibrin for two reasons: (i) Fibrin is used as the gold standard for the cultivation of LESCs and has been used for cultivating OMECs before transplantation [42] and (ii) Fibrin serves as a carrier to transfer cells to the transplanted eye surface [42]. This ensured that our study closely mirrored clinical conditions. Harvesting OMECs from fibrin glue using enzymes was necessary due to an experimental reason (obtaining a cell suspension after cell culture), but it is also consistent with the possibility of long-term storage of collected OMECs. This aspect is crucial for situations requiring repeated grafting. We, thus, demonstrated that OMECs can be removed enzymatically from a fibrin carrier without significant DNA damage.

The study’s limitation lies in the relatively low number of donors and the absence of primary (non-cultured) cells as controls. However, it is important to note that primary cells’ DNA damage levels are not directly relevant since only cultured cells attached to the proper substrate can be used for grafting. Within this limited cohort (7 donors), no differences in genotoxicity were observed based on donor age or sex. While some studies have shown that DNA damage measured by MNi test or comet assay may vary between men and women [43,44], a large-scale retrospective analysis of population studies using comet assay found no statistically significant differences between sexes [45]. Additionally, the background level of DNA breaks in human cells is typically around 10% tail intensity [45], consistent with our observations. The variations observed among subjects in this study remain within the expected physiological range. Moreover, each donor served as their own control, with cells cultivated in two different media, further minimizing potential confounding effects of age or sex differences. In conclusion, our results indicate that OMECs can be cultivated effectively in both COM and XF media without inducing significant DNA damage. It is a noteworthy discovery, especially considering that XF media are often favored in clinical settings due to their reduced risk of contamination and transmission of animal-derived pathogens. The ability to cultivate OMECs in XF media without compromising genomic integrity supports their use in clinical applications, enhancing the safety and effectiveness of ocular surface transplantation.

## Supplementary Material

geaf008_suppl_Supplementary_Materials

## Data Availability

No new data were generated in support of this publication.
