# Supplementary material for: DNA damage in oral mucosal epithelial cells cultured in complex and xenobiotic-free media: a comparison study
Source: Mutagenesis. 2025 Jun 28;40(3):526–32. doi: 10.1093/mutage/geaf008 (PMC12395244; doi:10.1093/mutage/geaf008)
Supplement: geaf008_suppl_Supplementary_Materials [file geaf008_suppl_supplementary_materials.docx]

**Supplementary Material**

**
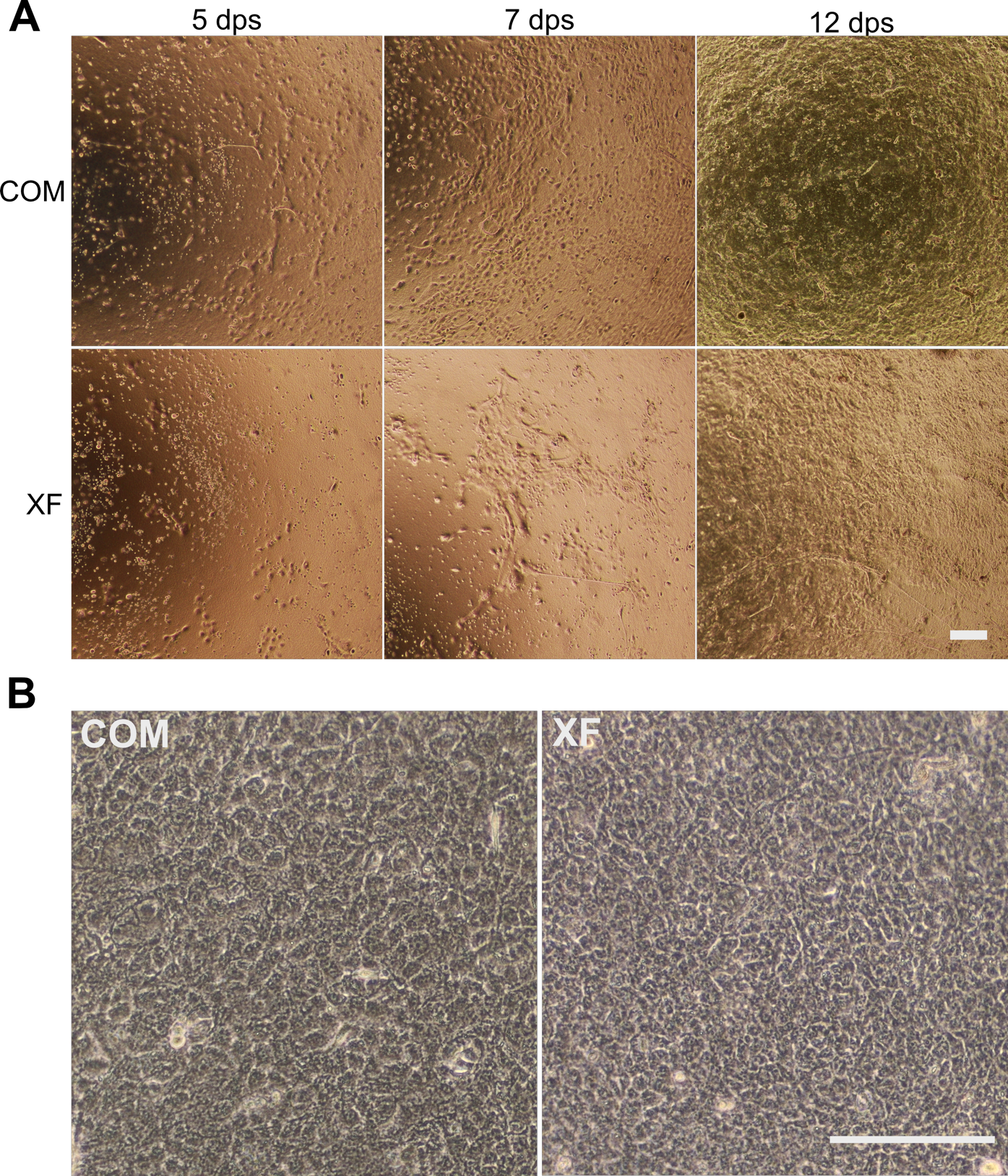
**

Figure: A. Inverted phase-contrast microscopy of cultures on fibrin complex media (COM) and fibrin xenobiotic-free media (XF), showing the progress of proliferation until confluence observed at 5 days post-seeding (dps), 7 dps, and 12 dps. B. The cobblestone-like morphology of cells in both culture media at confluence. Scale bars: 200 μm.
